# Supplementary material for: NorMASS: A normative MAS-based modeling approach for simulating incentive mechanisms of Q&A communities
Source: PLoS One. 2023 Feb 9;18(2):e0281431. doi: 10.1371/journal.pone.0281431 (PMC9910717; doi:10.1371/journal.pone.0281431)
Supplement: Attachment — Submitted filename: Response to Reviewers.doc [file pone.0281431.s001.doc]

Dear Reviewers,

Thank you for giving us the opportunity to submit a revised draft of the manuscript “NorMASS: A normative MAS-based modeling approach for simulating incentive mechanisms of Q&A communities” for publication in the Journal of PLOS ONE. We appreciate the time and effort that you and the reviewers dedicated to providing feedback on our manuscript and are grateful for the insightful comments on and valuable improvements to our paper. We have incorporated most of the suggestions made by the reviewers. Those changes are highlighted in the file labeled “Revised Manuscript with Track Changes”. Please see below, for a point-by-point response to the reviewers’ comments and concerns.



Best regards,

Yi Yang and Xinjun Mao.

**Reviewer #1**: I carefully read the manuscript “NorMASS: A normative MAS-based modeling approach for simulating incentive mechanisms of Q&A communities”, which proposed a Normative Multi-Agent System based Simulation approach to simulate community emergence.

This is a well written paper containing some interesting results. But there still are some shortcomings that need to be addressed：

1. This is a well-organized paper. However, I do not think it is necessary to divide it into 7 sections. For example, section 7 “Conclusion” could be integrated with section 5, and I suggest that the content of section 6 "Related Work" could be described in the section “Introduction”.

**Answer**: Thank you for your good advice. We integrated part of section 6 "Related Work" into the section "Introduction", adjusted section 6 “Related Work” to section 2, and integrated section 7 "Conclusion" with section 5.

2. The figures in the manuscript are quite small and not very clear, especially Figure 8 - Figure 11.

**Answer**: We have enlarged all the figures in the manuscript to ensure they are clear.

3. The equations in the manuscript are numbered from 3.3.1，however，I suggest that all the equations should be numbered, which means starting from subsection 3.2.3.

**Answer**: Thank you for your good advice. We have numbered all the equations in the paper.

**Reviewer #2**: The paper proposed a normative MAS-based approach for simulating incentive mechanisms. The work is very interesting, but I have a few small questions：

1、Could the authors consider comparing real data from multiple communities instead of simulated data?

**Answer**: Thank you for your good advice. We would like to make explanations regarding the usage of the simulated data.

First, it is rather difficult to obtain complete and real community data specifically for our research purposes. Thus, we designed a simulation system including a data generator, which provides a way to predict the effects of incentive mechanisms, even in the absence of real data.

Second, the simulated data can save time and effort on data gathering. Due to the large amount of users, it is time-consuming to gather the real data, which is not conducive to timely finding the potential problems of the incentive mechanism and adjusting the incentive mechanism design. For example, the SO community has more than tens of millions of users. To solve this problem, sampling is an appropriate method. The generation of simulation data can be regarded as a form of sampling, which generates the data as close as possible to the distribution of the real community. In the paper, we provide the distribution similarity between the two.

Third, the simulated data enables fast and efficient experiments. As shown in the figure below, the left is the experimental method adopted in this paper. On the right is the suggested method. It can be seen that both experimental results are compared to the emergence of the real community. Due to the use of the simulated data, our experimental method can achieve the desired effect faster and more efficiently than the suggested one.

2. Could you add more explanation to section 4.3

**Answer**: We have added some explanation to the section (section 5.3 in the revised manuscript) .

3. There are some obvious spelling mistakes in the article.

**Answer**: We have revised the manuscript carefully.
